# Treatment of trigeminal neuralgia by acupuncture combined with Chinese medicine from the perspective of modern medicine: A review

**DOI:** 10.1097/MD.0000000000040318

**Published:** 2024-11-01

**Authors:** Yue Liu, Dongyan Wang, Shenwei Li, Xu Dong, Jiajing Sun, Jingyi Li, Ying Zhang, Yixiao Han

**Affiliations:** a Heilongjiang University of Chinese Medicine, Harbin, Heilongjiang Province, China; b Second Affiliated Hospital of Heilongjiang Traditional Chinese Medicine, Harbin, Heilongjiang Province, China; c Department of Acupuncture, The First Affiliated Hospital of Wenzhou Medical University, Wenzhou Zhejiang Province, China.

**Keywords:** acupuncture, combination of acupuncture and medicine, depression and anxiety symptoms, pathophysiology, trigeminal neuralgia

## Abstract

Trigeminal neuralgia (TN) is characterized by recurrent episodes of transient severe pain in its distribution area, with abrupt onset and termination. With the progression of the disease, patients are prone to concurrent psychiatric disorders, such as anxiety and depression, which seriously affect patients’ quality of life. Currently, anticonvulsant drugs are commonly used in clinical practice as the primary treatment, but long-term use of drugs is prone to drug resistance, limiting clinical application. Acupuncture and traditional Chinese medicine (TCM), as alternative and complementary therapies, can make up for the deficiencies in modern medicine and are accepted by patients with the advantages of safety and effectiveness. TCM therapy works by promoting the release of endogenous opioid peptides, adjusting the level of inflammatory factors, and improving negative emotions to exert analgesic effects. This paper discusses the clinical efficacy and safety of acupuncture combined with Chinese medicine in the treatment of TN from the perspective of modern medicine and provides a theoretical basis for seeking better therapeutic targets.

## 1. Introduction

Trigeminal neuralgia (TN), also known as “painful twitching,” is a common chronic neuropathic pain syndrome, manifested by recurrent paroxysmal electric shock-like or knife-like sharp pain in the distribution area of the trigeminal nerve in the face.^[1]^ It is often caused by brushing teeth, eating, or cold stimuli, lasts for several seconds or minutes, and stops abruptly. Due to the extreme nature of the pain, patients experience profound events with varying frequency every day, which seriously affects the quality of life.^[2]^ TN is classified into 2 types: primary and secondary.^[3]^ In the former, the etiology is unknown, and it is induced by touching a trigger point with transient and severe pain, in the latter, it is secondary to multiple sclerosis and tumorous diseases of the base of the skull, and the pain is persistent and accompanied by neurological damage.^[4]^ The onset of TN is accompanied by tearing-like severe pain, which easily triggers anxiety and depression in patients and increases the burden on families and society.^[5]^

The treatment of TN is a hotspot of current research, mainly by surgical treatment and medical treatment.^[6]^ Surgery, as an effective treatment, has many limitations on patients’ physical fitness, many complications, the long-term efficacy is not ideal, and the treatment cost is high, as shown in the survey:^[7]^ in the last ten years, the management cost of TN in the United States has been more than $9,400,000. The long-term use of drugs may cause adverse reactions and develop drug resistance, and the recurrence rate of medical treatment of TN is 18.5%.^[8]^ Based on these limitations, there is a lack of ideal interventions in modern medicine. Some scholars have demonstrated the effectiveness of traditional Chinese medicine (TCM) in the treatment of TN through meta-analysis and sequential analysis, and compared with carbamazepine tablets, TCM was able to significantly relieve pain, and the incidence of adverse events was significantly lower.^[9]^ In this paper, based on the pathophysiological mechanisms of TN, we discuss the treatment of TN by acupuncture combined with Chinese medicine.

## 2. Epidemiology of trigeminal neuralgia

TN has become a global public health problem due to its recurrent severe pain and unpredictability. In a 2016 survey of adults in the U.S., the prevalence of TN was found to increase with age, with a higher prevalence of TN in women, up to 5.9/100,000 person-years, and it usually involves both the maxillary and mandibular branches.^[10]^ An epidemiological report on China^[11]^ showed that the prevalence of TN in China was 21.68/100,000 person-years. The National Health Insurance Service of Korea counted the medical data of 51,276,314 adults and found that the prevalence of TN in Korea was 100.21/100,000 person-years in 2018, with the highest prevalence in the age group of 51 to 59 years old, and the ratio of male to female patients was about 1:2.14.^[12]^ The higher prevalence in women than in men is due to gender differences in the way the brain responds to pain, and women are more capable of perceiving pain than men.^[13]^

Sathasivam^[14]^ found that the mean age of TN was 58.7 years, it occurred predominantly in females (61.6 percent), and the involvement of the right mandibular ramus was a characteristic feature of Asian patients, based on the statistics of Asian patients attending dental clinics. A Canadian government census of the population of Ontario found that TN affects 27/100,000 new patients per year and that TN is so painful that it is also known as a “suicide disease.”^[15]^ TN is usually disseminated, and familial TN has been poorly reported. Harris,^[16]^ whose series identified 1433 patients with TN, with familial aggregation of 30 cases, or approximately 2 percent, suggested that familial aggregation of TN is rarely mentioned, possibly because of inadequate history and information gathering.

## 3. Clinical features and complications of trigeminal neuralgia

Trigeminal neuralgia is a kind of peripheral neuropathy pain.^[17]^ The clinical manifestation of TN is an intolerable and severe pain in the distribution area of the trigeminal nerve in the unilateral facial area, with no premonition before the onset of the attack, and the patient is often accompanied by facial flushing, and even symptoms of a runny nose, drooling, and watering of the eyes. TN has a distinct point of tenderness in the facial area, which usually distributes in the eyebrow ridge bone, paranasal area, and corners of the mouth, and it can be caused by washing the face and brushing the teeth.^[18]^

Recurrent episodes of long-term chronic pain can have a certain impact on patients’ psychological health, prompting the body to produce a series of defense reactions, shortening the patients’ sleep time, and also producing some emotional disorders such as anxiety and depression, which limit social communication and daily activities.^[19]^ In addition, clinical studies have shown that there is an interaction between chronic pain and depressive states, which means that long-term chronic pain induces a depressive state, and the depressive state aggravates the patients’ perception of pain, also known as nociceptive hypersensitivity.^[20]^ Pain and depression develop together clinically manifesting similar alterations in neuroplasticity, and common brain dysregulation regions in depression include the anterior cingulate cortex and hippocampus, where gray matter volume loss is consistent with chronic pain-related brain region alterations.^[21,22]^ However, in clinical treatment, TN patients mostly complain of pain, and the correlation between depression, anxiety, and other emotions and pain is often ignored by doctors.^[23]^

## 4. Diagnosis of trigeminal neuralgia

The diagnosis of TN is mainly confirmed by taking a detailed history and relying on the patient’s clinical manifestations. Referring to the diagnostic criteria for TN published by the International Committee for the Classification of Headache in 2018: the patient’s face can induce pain in the area of the trigeminal distribution because of light touch, and this pain presents as a sharp, knife-like, recurring pain, and the neurological examination can be unremarkable.^[24]^ Diagnosing TN is extremely challenging due to the lack of laboratory tests or biomarkers for TN. But now, with the development of neuroimaging techniques, it offers the possibility to diagnose TN. The European Academy of Neurology recommends the use of a combination of 3 high-resolution sequences of magnetic resonance imaging (MRI) for the diagnosis of TN, and trigeminal reflexes as an alternative to MRI if the patient is unable to undergo the test because of contraindications to MRI.^[25]^

Cheng^[26]^ analyzed 60 patients with TN by high-resolution MRI technique to quantitatively assess the volume of the trigeminal nerve and found that the volume of the trigeminal nerve on the affected side was all significantly smaller than that on the healthy side. He concluded that the use of high-resolution MRI to assess the morphological changes of the trigeminal nerve can improve diagnostic accuracy. MRI helps in observing the vascular compression on the trigeminal nerve, it can be used to analyze quantitatively the vascular compression resulting in microstructural changes such as demyelination of nerve fibers and axonal rupture.^[27]^ In a study that included 30 patients with TN, the angle between the trigeminal nerve and the brain bridge was found to be acute in 25 patients with TN, suggesting a significantly higher chance of neurovascular compression of the trigeminal nerve.^[28]^ Therefore, regarding the diagnosis of TN, in addition to asking for medical history to exclude diseases that can be easily misdiagnosed, the neuroimaging technology of MRI can help us make a quick diagnosis and provide a reliable basis for the subsequent treatment plan.

## 5. Pathophysiological mechanisms of trigeminal neuralgia

### 5.1. Peripheral neuropathy theory

Some scholars have found mast cell degranulation on the trigeminal nerve branches of TN patients, which can lead to histamine release and aggravate nerve edema.^[29]^ From the point of view of anatomical structure, the right side of the human body’s foramen circumference and foramen ovale is relatively narrower than that of the left side, and the edematous nerves are subjected to entrapment by bony conduits at the base of the skull, which induce TN, and at the same time, it also explains the phenomenon that clinically, TN patients have more morbidity on the right side than that on the left side.^[30]^ Studies have suggested that the onset of TN is due to long-term vascular compression of the trigeminal nerve root, which in turn leads to demyelination.^[31]^ Clinical studies have found that some TN patients without vascular compression may experience demyelination, indicating that vascular compression is not necessarily the cause of TN.^[32]^ Devor^[33]^ first proposed the “ignition” theory, which suggests that short circuits occur between demyelinated axons and unmyelinated axons in the trigeminal nerve, and a slight stimulus can cause a short circuit. This theory suggests that short circuits occur between demyelinated and unmyelinated axons of the trigeminal nerve and that minor stimuli can aggravate the short circuits in neighboring nerve fibers, which repeatedly accumulate in nociceptive neurons, causing intermittent, episodic severe pain. The onset of TN is a very rapid process, and the “ignition” theory can vividly explain the nature of TN triggering, trigger point formation, and the spatial propagation of pain. It has also been suggested that the spontaneous discharge behavior of the afferent nerves of the trigeminal nerve causes excitation of the peripheral nerves, which induces paroxysmal discharges that result in pain.^[34]^

### 5.2. The central neuropathy theory

The central nervous system lesion theory suggests that the sensory deficits (severe pain or hyperalgesia) seen in patients with TN originate from trigeminal spinal tract nucleus lesions, brainstem, or cerebral cortex injury.^[35]^ TN is similar to sensory-like epileptic seizures, on the 1 hand, due to the fact that antiepileptic drugs, such as carbamazepine, can be effective in relieving TN, and on the other hand, some scholars have documented abnormal discharges of the trigeminal spinal tract nucleus in PET/CT scans of patients with TN, and all of the above can support the theory that trigeminal neuralgia belongs to epileptic seizures.^[36]^ Nguyen^[37]^ found that the use of electric current on the motor cortex of the central sulcus of the brain in TN patients could effectively relieve the pain condition of TN patients, and there were no epileptic seizures during the whole treatment process, thus indicating the involvement of the cerebral cortex in the pathogenesis of TN. With the development of medical imaging technology, more and more people are using functional MRI (fMRI) to explore the central mechanism of trigeminal neuralgia. One study^[38]^ found that during pain episodes in TN patients, pain stimulation led to increased activity in the somatosensory cortex, trigeminal nuclei, and thalamus, brain regions that are closely related to pain sensory processing. In addition, some scholars^[39]^ observed abnormalities in brain structure and function in TN patients by fMRI, which showed significant alterations in the functional connectivity of the frontal-limbic circuits, as well as a reduction in the gray matter volume of the anterior cingulate cortex and abnormalities in pain-related emotional responses in TN patients, compared with healthy individuals.

### 5.3. Immune-biochemical theory

Immune-biochemical factors are important factors affecting nerve demyelination. Macrophages, mast cells, and vascular endothelial cells were significantly increased in the demyelinated tissues of the trigeminal nerve, and biochemical parameters such as calcitonin gene-related peptide (CGRP), nitric oxide, and amino acids were altered in patients with TN.^[40,41]^ Chen^[42]^ found that he was able to reduce neuropathic pain and anxiety-like behavior in TN mice by intervening in TN mice with chronic injury to the distal infraorbital nerve, suggesting that Toll-like receptor 2-mediated neuroinflammation is 1 of the mechanisms involved in the pathogenesis of TN. The trigeminal nervous system contains a variety of pain-related neuropeptides, and some scholars^[43]^ suggested that the intranodal release of CGRP could regulate the neuronal transmission of pain signals, and he found that the expression of CGRP was significantly higher in female mice than in male mice by means of interventions, which explains the sex difference in TN and also provides new ideas for the mechanism of the pathogenesis of TN. In addition, some studies have shown^[44]^ that the severe pain during TN attacks may be related to the excessive release of substance P (SP), and the pain disappears with the weakening of SP, which speculates that SP is closely related to the pathogenesis of TN, suggesting that we should reduce the content of SP and CGRP when treating TN and block their binding to the corresponding receptors to treat TN fundamentally.

### 5.4. Ion channel theory

Voltage-gated sodium channels can selectively pass through sodium ions and control the discharge of incoming nerves, playing an important role in the pathogenesis of TN. It is known that voltage-gated sodium channels in mammals are classified into 10 subtypes ranging from Nav1.1 to Nav1.9, and these neurons remain relatively quiescent under normal conditions, upon peripheral nerve injury, the neurons become overexcited, leading to aberrant spontaneous activity, and the sodium channels are involved in the process of pain transmission.^[45]^ Studies have confirmed^[46]^ that Nav1.8 can trigger nociceptive hypersensitivity, and the occurrence of abnormal pain can be temporarily reversed by knocking out the Nav1.8 gene in mice. It has been suggested^[47]^ that pain is related to high-frequency nerve signaling, and the use of dependent sodium channel blockers, which block excitatory nerve conduction, can improve the therapeutic index of TN and achieve optimal safety and efficacy. In addition to sodium channels, intracellular calcium ions also play an important role in signal transduction. Cavα2δ1, as an isoform of the calcium channel α2δ, is closely related to neuropathic pain. Recent studies have demonstrated that an increase in Cavα2δ1 promotes aberrant excitatory synaptic formation and presynaptic excitatory neurotransmitter release, which sensitizes spinal neurons and leads to pain onset, and gabapentin can alleviate this nociceptive sensitization and provide new ideas for clinical treatment of TN.^[48]^

## 6. Cognition of trigeminal neuralgia in traditional Chinese medicine

TN does not have a corresponding name in TCM books and can be classified as “facial pain” or “cheek pain” based on symptoms. TCM categorizes the pathogenesis of TN into internal and external factors. The twelve meridians and the qi and blood of the 5 zang-organs and 6 fu-organs converge in the head and face. When the meridians of the head and face are invaded by external wind-evil, it is easy to cause poor circulation of qi and blood, blockage of meridians, and thus trigger TN, emphasizing the dominant role of external wind-evil in this disease.^[49]^ The main underlying causes are the long duration of the disease in TN patients, damage to qi and blood, insufficient supply of nutrients, depletion of liver fluid, and loss of nourishment of tendons and veins, resulting in pain and suffering.^[50]^ According to the above, TN is often caused by wind-evil, blood stasis, and deficiency, leading to blockages in the meridians and qi and blood, with the liver being the main site of the disease. Therefore, in clinical treatment, attention should be paid to clearing the liver and dispelling wind, implementing targeted treatment, unblocking meridians, harmonizing qi and blood, and promoting yin-yang balance.

## 7. Traditional Chinese medicine treatment of trigeminal neuralgia

### 7.1. Mechanism of acupuncture in treating trigeminal neuralgia

#### 7.1.1. Sensory dimensions

Opioid peptides are important analgesic substances in the brain, of which β-endorphin (β-EP) is widely involved in stress regulation and the body’s response to pain, and β-EP can reduce the excitability of pain receptors and the transmission of action potentials to reduce pain.^[51]^ The study has shown^[52]^ that acupuncture can activate opioid receptors in primary afferent nerve endings, inhibit the release of SP in afferent endings to inhibit central sensitization, adjust plasma levels of β-EP and SP. Some scholars^[53]^ observed the effect of acupuncture on serum β-EP and SP in TN patients and found that the pain symptoms of TN patients improved significantly after acupuncture treatment, the level of serum β-EP increased, the level of SP decreased, and the oxidative stress response was alleviated. Good clinical efficacy was achieved.

Acupuncture can regulate the expression of ion channel pain receptors, among which transient receptor potential vanillic acid 1 (TRPV1) is involved in the formation, transmission, and regulation of pain sensation in chronic neuropathic pain.^[54]^ A study has found that electroacupuncture can steadily and continuously alleviate pain hypersensitivity induced by paclitaxel, suggesting that its mechanism may be related to electroacupuncture inhibiting the elevation of TRPV1 in rat dorsal root ganglia, thereby alleviating paclitaxel-induced peripheral neuropathic pain, suggesting that electroacupuncture can be a potential therapy for treating chronic neuropathic pain.^[55]^ In addition, inflammatory factors such as tumor necrosis factor-α (TNF-α) and interleukin-6 (IL-6) can interact with sodium-calcium channels on cell membranes, thereby increasing neuronal excitability and leading to pain. Acupuncture can alleviate pain by regulating inflammatory factors.^[56]^ Studies have confirmed that acupuncture treatment can improve the visual analog score (VAS) of TN patients, reduce the levels of inflammatory factors such as TNF-α and IL-6, reduce the inflammatory response, and improve the life satisfaction index of TN patients.^[57]^

#### 7.1.2. Emotional cognitive dimensions

Pain is a complex physiological and psychological activity and is 1 of the common clinical symptoms; pain can lead to emotions such as anxiety and depression, and such negative emotions can also counteract to exacerbate pain.^[58]^ The mechanisms underlying the co-morbidity of pain and mood disorders are complex and involve neural circuits in specific regions of the brain, including the anterior cingulate cortex, amygdala, and hippocampus.^[59]^ Acupuncture has been found to improve neuropathic pain while also regulating mood, with a dual modulating effect on pain sensation and pain mood.^[60]^ Some scholars^[61]^ evaluated the clinical efficacy of acupuncture in the treatment of TN with anxiety and depression, and the results showed that the acupuncture group could significantly improve the degree of pain, anxiety and depression in patients with TN, with significant efficacy and rare adverse effects. Cong^[62]^ used a sciatica model to induce depressive-like behaviors and explored the effects of electroacupuncture on pain and depression, and found that electroacupuncture was able to reverse the down-regulation of the expression of brain-derived neurotrophic factor (BDNF) and 5-hydroxytryptamine (5-HT) in the anterior cingulate cortex and spinal cord of neuropathic pain rats, and at the same time modulate the level of cAMP-response element binding protein (CREB), which were all indicated above. The analgesic and antidepressant effects of electroacupuncture were achieved by modulating the CREB-5-HT/BDNF signaling pathway in the anterior cingulate cortex and spinal cord of rats.

The long-term recurrent episodes of TN can easily transform into another dimension of pain, with pain cognition as the main focus. TCM believes that for patients with pain perception, acupuncture treatment should focus on “regulating the mind,” and acupoints such as Baihui and Yintang should be selected to enhance the analgesic effect of acupuncture.^[63]^ Gao^[64]^ observed the effect of acupuncture on cognitive function in TN patients, and the results showed that compared with the sham control group, the acupuncture group had significantly reduced pain levels, improved cognitive function, and improved quality of daily life. Research suggests that acupuncture can inhibit cognitive dysfunction induced by TN, alleviate pain, and its mechanism may be related to the improvement of synaptic transmission efficiency and vacuolar degeneration in rat hippocampal neurons by acupuncture.^[65]^ Therefore, with the deepening of understanding of pain and the establishment of a “biological-social-psychological-environment” medical model, doctors should not only treat TN pain attacks but also pay attention to the impact of emotions such as depression cognition on pain, and focus on improving the overall quality of life of TN patients.

### 7.2. Practice of acupuncture treatment of trigeminal neuralgia

Wang Hui^[66]^ randomly divided 96 TN patients into 2 groups. The control group was treated with carbamazepine tablets, while the observation group was treated with acupuncture in addition to it. The selected acupoints were Hegu, Taichong, and Xiaguan. After the treatment course was completed, it was found that the simplified Macquarie Pain Questionnaire (SF-MPQ) score of the observation group significantly improved, the pain was reduced, and the incidence of complications in TN patients was significantly reduced. Moreover, the pharmacoeconomic indicators reflected that acupuncture treatment had good economic benefits.

Feng Zhaohuizi^[67]^ observed the effect of warm acupuncture with Hegu stabbing on the clinical efficacy and pain level of TN patients. 92 cases of TN patients were selected and divided into a western medicine group and an acupuncture group according to the random number table method, and the acupuncture group selected Xia Guan, Nei Ting, Hegu, Fengchi, Sibai, and Cheng Jiang acupoints. It was found that the VAS score of TN patients decreased and the pain level was significantly reduced after acupuncture intervention at the end of the treatment. Moreover, acupuncture treatment can reduce the level of SP in serum and increase the level of β-EP. The reason for this analysis is that acupuncture stimulates the local nerves of the organism, improves the blood circulation of the tissues, and transmits the excitation of the nerve fibers to the central nervous system, which then achieves the purpose of reducing the pain.

Gong Fei^[68]^ retrospectively analyzed the clinical data of 78 TN patients admitted to Benxi City Hospital of TCM from July 2018 to February 2020. The study found that the effectiveness of the Shuci combined with warm acupuncture for the treatment of TN was higher than that of conventional Western medicine, and after acupuncture stimulation of the A-shi point, Xiaguan, and Hegu points, the scores of VAS of TN patients were reduced, and the Pittsburgh Sleep Quality Index (PSQI) The scores improved, indicating that acupuncture treatment not only relieves pain but also improves sleep quality, which helps patients return to normal life rhythm and is worthy of clinical promotion and application.

In a meta-analysis,^[69]^ 58 randomized controlled trials were reviewed to evaluate the effectiveness and safety of acupuncture and moxibustion, electroacupuncture, fire acupuncture therapy and conventional drug therapy. Through the analysis of VAS and numerical rating scale, the results showed that after removing the highly heterogeneous literature, the therapeutic effect of electroacupuncture was significantly better than that of fire acupuncture therapy and conventional drug therapy. At the same time, the incidence of adverse reactions in the electroacupuncture group was significantly lower than that in the conventional drug group. Therefore, electroacupuncture is a relatively safe intervention for treating TN, effectively relieving pain and improving the daily quality of life of TN patients.

### 7.3. Traditional Chinese medicine prescription for trigeminal neuralgia

Li Shunxin^[70]^ used rat infraorbital nerve compression to establish an animal model of TN and observed the therapeutic effect of compound Lishao tablets (Radix Paeoniae Alba, Radix Puerariae Lobatae, Ramulus Uncariae cum Uncis, Salvia miltiorrhiza Bunge, White Chrysanthemum, Viticis Fructus, and Mentha canadensis Linnaeus) on rats with TN, and meanwhile preliminary explored the mechanism of the Chinese herbal medicine’s onset of action. In the study, 60 TN rats were randomly divided into a model group, a carbamazepine group, a low-dose group of compound quinoa peony tablets, a medium-dose group and a high-dose group, and were treated for 28 consecutive days. The facial pain of rats is severe, and the mechanical withdrawal threshold (MWT) will sharply decrease. From the MWT value, the high-dose group of compound Lishao tablets has a MWT value of 19.58, which is significantly improved compared to the 14.17 MWT value of the carbamazepine group; in terms of inflammatory cytokines, the serum levels of tumor necrosis factor-α (TNF-α) and interleukin-1β (IL-1β) in the high-dose group of compound Lishao tablets were significantly decreased in rats; In terms of the expression of CGRP in trigeminal ganglion and phosphorylated p38 mitogen activated protein kinase (p-p38 MAPK) in the striatum of the brain, the expression levels of CGRP and p-p38 MAPK in the high-dose group of compound Lishao tablets were significantly reduced compared with those in other groups; therefore, it is suggested that compound Lishao tablets can play a therapeutic role in TN, and the mechanism of which may be related to the inhibition of overexpression of inflammation factors, and down-regulation of CGRP in the trigeminal ganglion to reduce nociceptive transmission and nociceptive sensitization.

Zhang Fang^[71]^ designed a randomized controlled trial to investigate the effect of Quenching Wind and Penetrating Pain Relieving Soup on serum SP and β-EP in TN patients, 180 TN patients were divided into western medicine group (carbamazepine Tablets) and TCM group (Salvia miltiorrhiza Bunge, Ostrea gigas thunberg, Angelicae Sinensis Radix, Ramulus Uncariae Cum Uncis, Pheretima, Carthami Flos, Semen Persicae, Radix Glycyrrhizae and Scolopendra subspinipes) according to the method of randomized numerical table, and the results showed that the VAS scores and PSQI scores of the TCM group were significantly lower than those of the western medicine group, and the Chinese medicines had advantages in relieving pain and improving sleep, while at the same time the SP level decreased and the β-EP level increased in TN patients after Chinese medicine treatment, suggesting that the Chinese medicines exerted their therapeutic effects by adjusting the release of pain mediators.

Qiao Xiaojun^[72]^ observed the clinical efficacy of mouse nerve growth factor combined with self-prepared Jinling Tongjiao formula on postoperative TN patients. Patients were randomly divided into 2 groups, both of which were subjected to microvascular decompression surgery; the control group was not given any treatment, while the observation group was given mouse nerve growth factor combined with self-prepared Jinling Tongjiao formula (Radix Paeoniae Alba, Rhizoma Chuanxiong, Semen Persicae, Radix Angelicae Dahuricae, Boswellia sacra, and Commiphora myrrha) to treat TN patients. VAS, PSQI, and Chinese medicine evidence scores of the 2 groups were observed before and after treatment, and it was found that the scores of the observation group improved significantly, and the long-term follow-up was conducted after 6 months, and it was found that the recurrence rate of the observation group was much lower than that of the control group, and the analysis of the reasons may be related to the fact that the murine nerve growth factor combined with the Jinling Tongjiao formula adjusted the concentration of inflammatory cytokines and vasoactive intestinal peptide in serum and made the concentration of the above indexes tend to be normal, which shortened the time of TN and reduced the degree of pain. Therefore, post-surgery adjuvant neurotrophic drugs and Chinese herbal formulae have obvious advantages in the treatment of TN.

### 7.4. Treatment of trigeminal neuralgia by acupuncture combined with traditional Chinese medicine

Xu Dan^[73]^ investigated the effects of acupuncture combined with Chuanxiong Chatiao San on pain and sleep in TN patients in a randomized controlled trial in 2023, in which 90 TN patients were randomly assigned to the control group and the observation group according to the principle of 1:1. The control group was given western medicine gabapentin treatment, and the observation group was given acupuncture combined with TCM on its basis, and the TCM chose Chuanxiong Chatiao San (Rhizoma Chuanxiong, Radix Angelicae Dahuricae, Schizonepetae Herba, Radix Saposhnikoviae, Mentha canadensis Linnaeus, Radix et Rhizoma, Semen Persicae, Carthami Flos, Scorpio, and Radix Glycyrrhizae), together with acupuncture selecting Cuanzhu, Yuyao, Taiyang, Sibai, Daying, and Xiaguan acupoints for external treatment. The results showed that after acupuncture combined with TCM treatment, the simplified McGill Pain Questionnaire-2 (SF-MPQ-2) score and PSQI score of TN patients were significantly reduced, which improved the sleep quality of patients by reducing the degree of pain, alleviating negative emotions such as depression. In addition, it was found that after acupuncture combined with TCM treatment, the inflammatory factor indicators of TN patients were significantly improved. This can be achieved by increasing the pain threshold or inhibiting pain stimuli, thereby reducing the release of related pain factors.

Huang Zhenzhen^[74]^ investigated the effects of Shugan Tongjiao formula (Radix Paeoniae Rubra, Radix Paeoniae Alba, Radix Angelicae Dahuricae, Rhizoma Chuanxiong, Citri Reticulatae Pericarpium, Radix Bupleuri, Aurantii Fructus, Radix Glycyrrhizae, Carthami Flos, Scorpio, and Scolopendra) combined with needling (Hegu, Taichong, Neiting, Sibai, Xiaguan, Dicang, and Cuanzhu) on the degree of pain as well as the level of neurological substances of patients with TN. The study randomly divided 90 patients into a control group and an observation group, with 45 patients in each group. The control group was given conventional treatment, and the observation group was based on the control group and treated with Shugan Tongjiao formula combined with acupuncture, comparing the VAS scores, TCM evidence scores, SP, β-EP, and CGRP levels of TN patients before and after the treatment. Acupuncture combined with TCM not only effectively relieves the pain symptoms of the patients and slows down the frequency of the pain episodes, but also adjusts the level of neurotoxic substances and improves the quality of life.

Yan Gaigxia^[75]^ designed a randomized controlled trial using a simplified McGill scale to evaluate the effect of acupuncture combined with TCM on patients with TN. The study randomly divided patients with TN into 3 groups: 24 patients in the TCM group were treated with a self-prescribed formula (Radix Bupleuri, Pinelliae Rhizoma, Ginseng Radix et Rhizoma, Radix Glycyrrhizae, Radix Scutellariae), and 25 patients in the acupuncture group were treated with (Cuanzhu, Sizhukong, Sibai, Dicang, and Jiache) external needling. The other 25 patients received acupuncture combined with medication. The results showed that TN patients had different degrees of fear and dread before treatment, and at the end of the treatment, compared with the other 2 groups, the acupuncture-medicine combination group significantly increased the pain threshold of TN patients, reduced the number of pain episodes, soothed the bad mood, and improved the quality of life. Moreover, the follow-up of TN patients revealed that the clinical efficacy of acupuncture combined with herbal medicine in treating TN patients was durable and stable. The detailed evidence of TCM treatment for TN is shown in Table 1.

**Table 1:** Evidence for the treatment of TN with traditional Chinese medicine.

Experimental researchers	Group and intervention	Result	Conclusion
Wang Hui^[66]^	Patients diagnosed with TN (n = 96) The patients were divided into an observation group and a control group by the blind selection method; the control group was treated with carbamazepine tablets, and the observation group was treated with acupuncture in combination with the control group for 3 mo.	The total clinical effective rate of the observation group was 97.92%, which was higher than 81.25% of the control group; the total complication rate of the observation group was 8.33%, which was lower than 25.00% of the control group; and the total cost of treatment of the observation group was lower than that of the control group, and the differences were all statistically significant (*P* < .05).	Combined acupuncture can effectively improve the clinical outcome of patients with trigeminal neuralgia, improve pain symptoms, reduce the incidence of patient complications, and have better pharmacoeconomic benefits.
Feng Zhaohuizi^[67]^	Patients diagnosed with TN (n = 92) were divided into Western medicine group (single) and acupuncture group (double) according to the single and double numbers of outpatient clinics, and were treated continuously for 3 wk.	After 3 wk of treatment, the VAS scores, TCM evidence points and serum SP levels of both groups were lower than those before treatment, and they were all lower in the acupuncture group than in the western medicine group; moreover, the SP levels of the acupuncture group were lower than those of the western medicine group, and the β-EP levels of the acupuncture group were higher than those of the western medicine group, and the differences were statistically significant (*P* < .05).	Acupuncture treatment of patients with TN improves clinical efficacy, reduces the degree of pain in patients, and reduces serum SP levels and increases β-EP levels.
Gong Fei^[68]^	Patients confirmed as TN (n = 78) were randomly divided into 2 groups, the control group was given conventional Western medicine treatment, and the observation group was treated with acupuncture for 3 wk.	VAS scores and scores of sleep quality, sleep duration, sleep efficiency, and daytime dysfunction in the observation group were significantly lower than those in the control group, and the difference was statistically significant (*P* < .05).	Acupuncture is effective in treating patients with TN, improving their clinical symptoms, relieving pain, and improving their sleep quality.
Yin Z^[69]^	58 randomized controlled trials involved 4126 participants diagnosed as TN who received acupuncture and moxibustion, electroacupuncture, fire acupuncture and conventional drug treatment.	Compared with conventional drug treatment, the VAS score and NRS score of electroacupuncture therapy were significantly improved, the total effective rate and cure rate were significantly increased, and the incidence of adverse reactions was significantly reduced.	Electroacupuncture is the best, followed by acupuncture and moxibustion and fire acupuncture, all of which are better than western medicine alone. Due to the bias in the quantity and quality of the included studies, more relevant research is needed in the future to further confirm.
Li Shunxin^[70]^	The TN rat model was randomly divided into model group, carbamazepine group and low, medium and high-dose (420, 840, and 1680 mg/kg) groups of compound Lishao tablets, which were treated continuously for 4 wk.	Compared with the carbamazepine group, rats in the high-dose group of compound Lishao tablets showed a significant increase in MWT (*P* < .05) and a significant decrease in the levels of TNF-α and IL-1β in serum and trigeminal ganglion, as well as the expression of p-p38 MAPK in the striatum (*P* < .05).	The mechanism of the therapeutic effect of compound Lishao tablets in patients with TN may be related to the reduction of the expression of inflammation-related factors as well as proteins.
Zhang Fang^[71]^	Patients diagnosed with TN (n = 180). Random number table method were divided into western medicine group (carbamazepine tablets) and traditional Chinese medicine group (Xifeng Tongluo formula) and treated for 4 wk.	Compared with the western medicine group, the decrease in SP level was greater and the level of β-endorphin was elevated in the traditional Chinese medicine group (*P* < .05), and the VAS and PSQI scores in the traditional Chinese medicine group were lower than those in the western medicine group after treatment (*P* < .05).	Xifeng Tongluo formula can reduce the release of pain mediators, relieve pain, improve sleep quality, and is worth promoting the use of clinical application.
Qiao Xiaojun^[72]^	Patients diagnosed with TN (n = 94) were randomly divided into 2 groups, the control group was not given any treatment, and the observation group was given mouse nerve growth factor combined with self-prepared Jinling Tongjiao formula for 8 wk.	The VAS, PSQI, VIP and TCM symptom scores of the observation group were lower than those of the control group (*P* < .05), and the 5-HT and β-EP were higher than those of the control group, and the relapse rate of the observation group was lower than that of the control group at 6 mo of treatment (*P* < . 05).	Combined with the self-formulated Jinling Tongjiao formula, the treatment can effectively relieve pain, improve patients’ quality of life, and reduce the recurrence rate.
Xu Dan^[73]^	Patients diagnosed with TN (n = 90) The patients were randomly divided into 2 groups: the control group was given gabapentin tablets, and the observation group was treated with acupuncture combined with Chuanxiong Chatiao San for 20 d on the basis of the control group.	The SF-MPQ-2 score, PSQI score and inflammatory factor indexes of the observation group were significantly lower than those of the control group (*P* < .05); the total clinical effectiveness rate of the observation group was higher than that of the control group (*P* < .05).	Acupuncture combined with Chuanxiong Chatiao San could modulate the inflammatory response in TN patients and was significantly more effective than gabapentin tablets alone in improving pain and sleep.
HuangZhenzhen^[74]^	Patients diagnosed with TN (n = 90). The patients were randomly divided into 2 groups: the control group was given conventional treatment, and the observation group was given acupuncture combined with Shugan Tongjiao formula on the basis of the control group for 2 mo.	The VAS and TCM evidence scores of the observation group were lower than those of the control group; the levels of CGRP and SP were lower than those of the control group; and the level of β-EP was higher than that of the control group, and the difference was statistically significant (*P* < .05).	Acupuncture and moxibustion combined with Shugan Tongjiao formula treatment can effectively reduce the pain level of TN patients, improve their neurological substance level, and alleviate clinical symptoms.

5-HT = 5-hydroxytryptamine, β-EP=β-endorphin, CGRP = calcitonin gene-related peptide, IL-1β=interleukin-1β, IL-6 = interleukin-6, MWT = mechanical withdrawal threshold, NRS = numerical rating scale, p-p38 MAPK = phosphorylated p38 mitogen activated protein kinase, PSQI = Pittsburgh sleep quality index, SF-MPQ-2 = short-form McGill pain questionnaire-2, SP = substance P, TCM = traditional Chinese medicine, TN = trigeminal neuralgia, TNF-α=tumor necrosis factor-α, VAS = visual analogue score, VIP = vasoactive intestinal peptide.

## 8. Conclusion

Patients with TN are characterized by recurrent severe pain in the area of trigeminal innervation of the face, which severely affects the quality of life of patients and also constitutes a major burden on the health care system.^[76]^ Compared with carbamazepine tablets, the combination of acupuncture and Chinese medicine plays a key role as a safe and effective alternative therapy in reducing pain and recurrence rates, as well as minimizing side effects in TN patients.^[77]^ Due to the unpredictability of pain, TN patients are prone to concurrent anxiety and depression negative emotions. Clinicians should pay attention to the treatment of pain-related complications along with analgesia in the treatment of TN in order to achieve good clinical efficacy.

However, there are some problems in current research. Firstly, most studies focus on the effectiveness of TCM in treating TN, limited to clinical practice, and there is relatively little research on the mechanisms involved; secondly, there is no standardized criterion regarding the depth of acupuncture points, acupuncture manipulation, and the type of Chinese herbal medicine evidence; thirdly, a large number of studies focus on the effect during the treatment period, and few follow-ups are carried out.

Therefore, animal experiments should be conducted appropriately in future studies to explore the possible mechanisms of the needling effect; using visualization techniques such as ultrasound to standardize the depth and manipulation of acupuncture points; utilizing artificial intelligence to enhance TCM data mining and TCM assisted diagnosis, organizing the treatment experience of renowned doctors, and exploring the medication patterns for diseases; paying attention to the establishment of patient medical records, utilizing cloud follow-up platforms, and observing the long-term therapeutic effects of patients after treatment; In addition, psychological suggestion can cause complex neurological responses in acupuncture and TCM treatment, and how to rule out the placebo effect triggered by psychological cues deserves in-depth exploration by researchers.

## Author contributions

**Conceptualization:** Yue Liu.

**Data curation:** Yue Liu, Dongyan Wang, Shenwei Li, Xu Dong, Jiajing Sun, Jingyi Li, Ying Zhang, Yixiao Han.

**Formal analysis:** Yue Liu.

**Funding acquisition:** Yue Liu.

**Investigation:** Yue Liu.

**Methodology:** Yue Liu.

**Project administration:** Yue Liu.

**Resources:** Yue Liu.

**Software:** Yue Liu.

**Supervision:** Yue Liu.

**Validation:** Yue Liu.

**Visualization:** Yue Liu.

**Writing – original draft:** Yue Liu.

**Writing – review & editing:** Yue Liu.
